# Renal Sinus Lipomatosis in Transplanted Kidneys: An Unusual Clinical Case

**DOI:** 10.1155/2011/161759

**Published:** 2012-01-04

**Authors:** Luca Apicella, Gianfranco Vallone, Sossio Vitale, Gianluca Garofalo, Luigi Russo, Riccardo Gallo, Stefano Federico, Massimo Sabbatini

**Affiliations:** ^1^Nephrology and Renal Transplantation, Department of Systematic Pathology, University of Naples “Federico II”, 80131 Naples, Italy; ^2^Department of Radiology, University of Naples “Federico II”, Via S. Pansini 5, 80131 Naples, Italy

## Abstract

Renal sinus lipomatosis (RSL) represents an abnormal proliferation of the adipose tissue surrounding the renal pelvis of uncertain origin, associated with aging, obesity, steroid excess, infections, and calculosis. It represents a rare complication in transplanted kidneys, and, despite the accurate and prolonged radiological followup of transplanted organs, only a few cases of RSL have been described in graft recipients, with no remarkable effects on renal function. The diagnosis relies on ultrasonography (US), magnetic resonance imaging (MRI), computed tomography (CT), and, finally, percutaneous biopsy. We describe the case of an extensive RSL in a 38-year-old renal transplant recipient, diagnosed by ultrasonography and computed tomography. The patient underwent a radiologic study because of an acute, asymptomatic renal impairment, that led to the diagnosis of a RSL of unusual dimensions, associated with a discrete hydronephrosis. Paradoxically, after a short course of steroids, the recovery of renal function and the partial resolution of calyceal dilatation were observed. The rarity of this affection, the need of a differential diagnosis with fat-containing tumors, and the possibility of parenchymal inflammation associated with RSL, potentially responsive to steroids, are also discussed.

## 1. Introduction

The normal renal sinus contains a small quantity of fat that envelops the pelvis and the surrounding structures of the perinephric space and that may gradually increase with age and obesity. Renal sinus lipomatosis (RSL) represents a benign condition in which the perirenal fat proliferates to a variable degree around the kidney, the ureter, and the intrarenal collecting system, but does not lead to symptoms or renal impairment [1]. An abnormal proliferation of sinus fat may also occur with increased exogenous administration or endogenous production of steroids [2] and is also associated with processes causing the atrophy of renal tissue; in this latter case, it is defined renal replacement lipomatosis (RRL) [1, 3]. Indeed, RRL represents the extreme form of RLS in which the abnormal growth of perirenal fat is secondary to the severe renal destruction that follows recurrent urinary tract infections and/or calculus disease with hydronephrosis.

RLS is a relatively rare condition in normal kidneys and is even less common in renal transplanted kidneys. The presence of RSL in a renal transplant recipient was described for the first time in 1979 [4], and, since then, only three additional cases have been mentioned in 2005 [5], although described as RRL. We report a peculiar clinical case of RSL, characterised by a huge dimension and by acute renal impairment, never described to date, with recovery of renal function after steroid treatment.

## 2. Case Report

A 38-year-old man received a renal transplantation from cadaver donor on October 1999, after one year spent on dialysis. The early clinical course was characterised by a delayed graft function and two clinical episodes of acute rejection, both treated with steroid pulse therapy. The immunosuppressive treatment consisted of basiliximab, methylprednisolone, cyclosporine, and azathioprine (AZA). At discharge, creatinine plasma concentration was 2.0 mg/dL and remained stable thereafter. On October 2002, AZA was replaced by sirolimus, and some months later cyclosporine was completely withdrawn. No rejection episode, nor infectious problems were observed during the following years.

In 2007 the patient, due to work problems, moved to a different hospital for his periodic followup. On October 2010, routine laboratory analysis showed the asymptomatic rise of plasma creatinine to 3.2 mg/dL. An abdominal ultrasonography (US) was performed, showing a hyperechoic solid renal mass that completely enveloped kidney structures. The nephrologist suspected a renal neoplasia and advised the patient to contact us for a deeper clinical evaluation.

**Figure 1:**
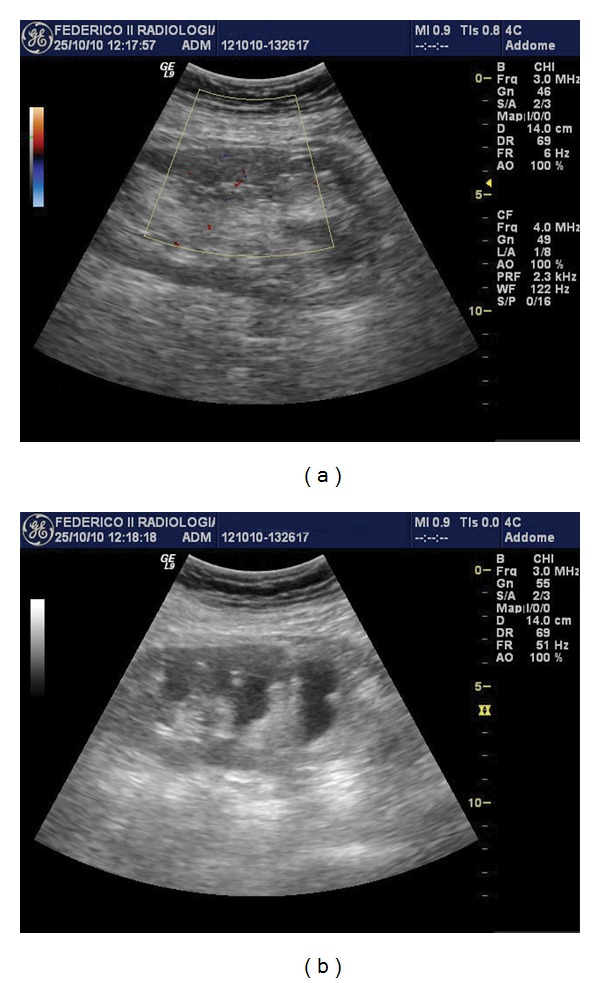
(a) US showing the highly echogenic appearance of renal sinus with fat tissue that covers most of renal surface. (b) Longitudinal scan of the transplanted kidney showing a discrete degree of hydronephrosis, more pronounced in the upper calyces.

At admission in our unit, the decline of GFR was confirmed (plasma creatinine: 4.2 mg/dL); palpation of the transplanted kidney evoked no pain, and diuresis was maintained. The US confirmed the alteration of renal structure due to the presence of a conspicuous nonvascularized mass (10.4 × 4.1 cm) that completely wrapped the kidney around, but had the characteristics of fat tissue, with a concomitant hydronephrosis (Figures 1(a) and 1(b)). A RSL was hypothesized, and a computed tomography (CT) scan was performed, since magnetic resonance imaging (MRI) could not be performed due to the presence of abdominal metallic clips. CT (with no contrast medium) confirmed the presence of the extensive fat proliferation that completely covered renal pelvis; the ureter was not visualized, nor calculi could be demonstrated in any scan.

Three different diagnoses were considered to explain the acute renal impairment: a mass effect of RSL on renal pelvis or an asymptomatic ureteral calculosis, both potentially determining hydronephrosis; third, a late acute rejection of the graft, with no peculiar echographic sign. Accordingly, we consulted the urologist about the possibility of ureteral stenting and suggested to the patient to perform a biopsy of both fat and renal tissue to exclude a neoplasia or a rejection. The patient refused these procedures. In the meantime, considering the clinical suspicion and what had been reported in a previous experience [5], we empirically decided to increase the dosage of methylprednisolone from 4 to 16 mg/day, postulating an acute inflammation either of the perirenal fat (of unknown origin) or of the ureter (passage of a renal stone?) able to determine a functional obstruction. Unexpectedly, 2 days after the changes in steroid treatment, plasma creatinine started to decrease (3.8 mg/dL), and in the following week its value reached 2.0 mg/dL, that is, that commonly observed in the previous years.

**Figure 2:**
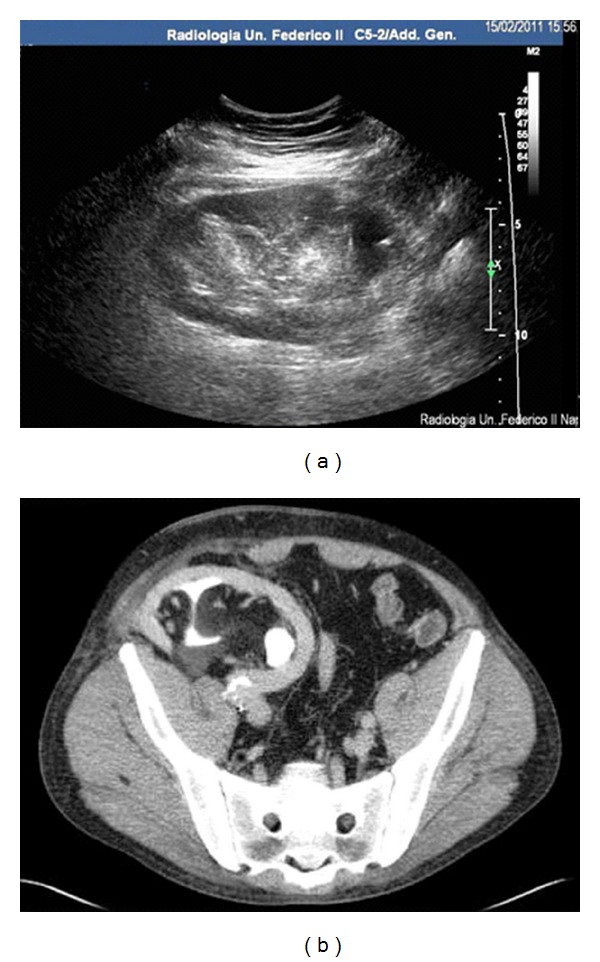
(a) US and (b) CT scan (with contrast medium) performed after the resolution of acute renal impairment, showing the persistence of a mild dilatation of upper calyces and the impregnation with contrast media of pelvicalyceal structures. The entity of perirenal fat is not modified.

After stabilisation of renal function, an US and a new CT (with contrast medium) were performed on December 2010, that showed the persistence of a slight dilatation in the upper calyces (localized hydrocalicosis) and the normal diameter of proximal ureter (Figures 2(a) and 2(b)). An interesting additional finding was the presence of contrast medium inside the wall of renal pelvis and the proximal portion of the ureter, a marker of chronic inflammation of these structures.

On October 2011, no change was observed in renal US nor in renal function, during the last follow-up visit of the patient in our unit.

## 3. Discussion

The discovery of RSL represents an uncommon finding of renal US either in normal subjects and in transplant recipients; indeed, there are just two papers describing RSL or RRL in these latter patients [4, 5], despite the accurate and prolonged radiological followup of transplanted organs. At difference with previous reports, here we describe a peculiar case of RSL characterised by its huge dimension, the onset of acute renal dysfunction, and its “paradoxical” resolution.

Acute renal impairment due to mass effect has never been described in previous patients with RSL or RRL, since fat insinuation around the renal pelvicalyceal system mostly determines localized hydrocalicosis. In our case, conversely, US clearly demonstrated a diffuse hydronephrosis, that partially disappeared after the recovery of renal function, since a mild dilatation still persisted in the superior calyceal group.

The cause of the acute renal failure is not clear; the degree of hydronephrosis, in fact, was not dramatic, and renal/ureteral stones could not be detected with US, nor with CT; moreover, the patient had no symptom (pain, dysuria, fever) recalling renal calculosis, although transplant recipients commonly do not have colic pain. An “a posteriori” diagnosis of late acute rejection was highly improbable considering that the dosage of steroids was inadequate to cure a rejection, although it is not possible to exclude that steroid treatment determined a beneficial effect in treating a mild, asymptomatic graft rejection. Unfortunately, the patient's refusal of renal biopsy prevented a definite diagnosis.

The strict temporal association between steroid administration and the recovery of renal function, however, allows to hypothesize that steroid administration had somehow resolved an unknown inflammation of the pelvis and/or of the ureter, determining the partial resolution of hydronephrosis and the return of plasma creatinine to its old value.

It should be stressed, however, that since there is no direct evidence that RLS was responsible for obstructive nephropathy and that only minor changes were observed in US after renal recovery, the hypothesis and the role of steroids remain speculative.

The beneficial effect of steroids seems paradoxical since these drugs are considered pathogenic factors of RSL [2, 4]; this assumption, however, should be reconsidered, given the rare occurrence of RSL in graft recipients who assume steroids even for decades. Indeed, the companion transplanted kidney shows no sign of RSL, despite a similar cumulative load of steroids, with exception of pulse treatments (no rejection episode). Therefore, the described association between RSL and pulse therapy of rejections could simply reflect a higher degree of inflammation. A study of Laouad et al. describes 3 cases of RSL/RRL characterised by the presence of delayed graft function, recurrent urinary tract infections, and multiple rejection episodes, all proinflammatory episodes [5]. Whether other factors are involved in determining RSL beyond chronic inflammation remains obscure: a role for sirolimus in our patient, however, seems unlikely considering the well-known antiproliferative properties of such drug; at present no further report of RLS associated with sirolimus treatment has been published.

In patients with RSL or RRL, a differential diagnosis with fat-containing neoplasms, like lipoma, angiomyolipoma, and, mostly, liposarcoma should be performed. Indeed, liposarcoma is usually located peripherally between the kidney and renal capsule and does not alter renal parenchyma; rare cases of liposarcoma originating within the renal sinus are easily identified at CT for the splay of renal parenchyma and the presence of internal thick soft tissue strands or nodules. Nevertheless, the percutaneous biopsy of RLS (also associated to renal biopsy) should be mandatory in transplant recipients, prone to develop any kind of neoplasia, since it represents the only possibility to have a definite diagnosis.

In conclusion, RSL is a rare and benign condition in renal transplant recipients that may impact renal function either acutely or in the long term, mostly when infections or calculosis ensue; a careful followup of this condition is advisable to prevent the shift of RSL to RRL, by treating inflammation and for a closer surveillance to avoid the development of fat-containing neoplasms.
